# Natural variation in host feeding behaviors impacts host disease and pathogen transmission potential

**DOI:** 10.1002/ece3.9865

**Published:** 2023-03-07

**Authors:** Alaina C. Pfenning‐Butterworth, Rachel E. Vetter, Jessica L. Hite

**Affiliations:** ^1^ School of Biological Sciences University of Nebraska Lincoln Nebraska USA; ^2^ Department of Botany University of British Columbia Vancouver British Columbia Canada; ^3^ Department of Pathobiological Sciences University of Wisconsin Madison Wisconsin USA

**Keywords:** anorexia, *Daphnia dentifera*, hyperphagia, illness‐mediated feeding behavior, *Metschnikowia bicuspidata*, resistance, tolerance

## Abstract

Animals ranging from mosquitoes to humans often vary their feeding behavior when infected or merely exposed to pathogens. These so‐called “sickness behaviors” are part of the innate immune response with many consequences, including avoiding orally transmitted pathogens. Fully understanding the role of this ubiquitous behavior in host defense and pathogen evolution requires a quantitative account of its impact on host and pathogen fitness across environmentally relevant contexts. Here, we use a zooplankton host and fungal pathogen as a case study to ask if infection‐mediated feeding behaviors vary across pathogen exposure levels and natural genetic variation in susceptibility to infection. Then, we connect these changes in behavior to pathogen transmission potential (spore yield) and fitness and growth costs to the host. Our results validate a protective effect of altered feeding behavior during pathogen exposure while also revealing significant variation in the magnitude of this response across host susceptibility and pathogen exposure levels. Across all four host genotypes, feeding rates were negatively correlated with susceptibility to infection and transmission potential. The most susceptible genotypes exhibited either strong anorexia, reducing food intake by 26%–42%, (“Standard”) or pronounced hyperphagia, increasing food intake by 20%–54% (“A45”). Together, these results suggest that infection‐mediated changes in host feeding behavior—which are traditionally interpreted as immunopathology— may in fact serve as crucial components of host defense strategies and warrant further investigation.

## Graphical abstract

Animals often vary their feeding behavior when infected or merely exposed to pathogens; this is part of the innate immune response and has many consequences. Here, we contribute to the understanding of the role feeding behavior plays in host defense and pathogen evolution by quantifying its impact on host and pathogen fitness across environmentally relevant contexts. Our results suggest that infection‐mediated changes in host feeding behavior may in fact serve as crucial components of host defense strategies.

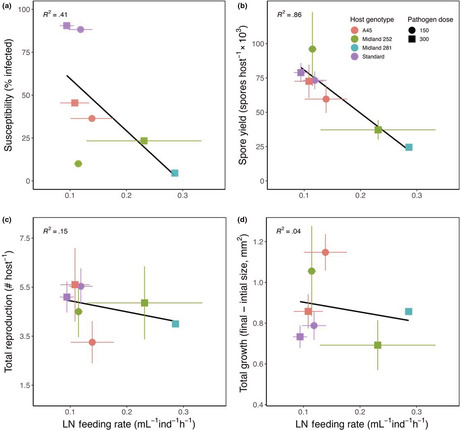

## 1 INTRODUCTION

Why do some individuals drastically decrease or increase their food intake when exposed to or infected by pathogens? This central question continues to puzzle both disease biologists and evolutionary epidemiologists. Beyond basic biology, answers to these questions have important applications for disease management in livestock and inform best practices for animal health and food security; food withdrawal prior to transport is standard in all livestock (Acevedo‐Giraldo et al., 2020), reduced food intake is considered a pre‐clinical sign of infection and often catalyzes medical interventions and drug treatments (Kanra et al., 2006; Schutz et al., 2014). Understanding when, why, and how food intake enhances or suppresses pathogen growth and transmission is, therefore, an important research objective (Ayres & Schneider, 2009; Cumnock et al., 2018; Kyriazakis et al., 2009).

Prominent hypotheses for these so‐called “sickness behaviors” posit that reduced food intake (“immune‐mediated anorexia”) is immunopathological but can function in pathogen avoidance or be manipulated by pathogens to increase transmission (Rao et al., 2017; Rogers & Bates, 2007). Increased food intake (“hyperphagia”), on the other hand, is assumed to enhance energetically costly immune functions (Dick et al., 2010; Giles, 1987; Ponton et al., 2011). Relative to immune‐mediated anorexia, hyperphagia during pathogen infection is less documented and the two behaviors are rarely discussed simultaneously (Hite, Pfenning, & Cressler, 2020). Taken together, however, these ideas suggest that in certain cases, the need for pathogen avoidance should outweigh the immune costs (and vice versa), highlighting that these behaviors should vary over relevant environmental contexts such as pathogen exposure levels or host genetic variation in susceptibility to infection.

To date, most empirical work on immune‐mediated changes in feeding behavior has generally taken a standard reductionist approach, focusing on immune stimulants (e.g., lipopolysaccharide) or single “LD‐50” doses. These studies also tend to focus on the molecular and physiological drivers and few studies have quantitatively linked changes in feeding behavior to host fitness or pathogen transmission potential (Hite, Pfenning, & Cressler, 2020). While these studies have certainly advanced the field, results remain mixed and no broad‐scale patterns have emerged. Inconsistent results across various host–pathogen systems, diverse pathogen genotypes, and differing infection routes challenge our understanding of the functional implications of these ubiquitous behaviors (Hite, Pfenning, & Cressler, 2020; Pike et al., 2019).

One reason for these variable responses is the immense diversity of genetic‐based traits that modulate immune responses in host populations. While the importance of host variation in immune defense is widely acknowledged, we know surprisingly little about how this variation affects host disease and pathogen fitness. Moreover, this diversity is not well represented in the few inbred lab lines typical of model systems routinely used to study immune defense (Duffy et al., 2021; Graham, 2021). Changes in feeding behavior represent one axis in the defense arsenal a host enlists. The particular suite of defenses will vary, contingent on genetic variation in, for example, susceptibility to infection, current environmental conditions, or pleiotropic effects related to other life‐history traits (Moret & Schmid‐Hempel, 2000; Schulenburg et al., 2009). Therefore, to gain a general understanding of the role that illness‐mediate feeding behaviors play in host–pathogen biology, we must account for the genetic diversity and environmental complexity present in the natural settings where hosts encounter pathogens.

As a first step in this endeavor, we use host genotypes sampled from nature to explicitly focus on the net effects of changes in illness‐mediated feeding behaviors. Specifically, we examine links among changes in feeding behavior, host growth and reproduction, and parasite fitness (within‐host spore load). Our work here was prompted by our identification that genotypes of the focal zooplankton host, *Daphnia dentifera*, vary in susceptibility to a fungal pathogen, *Metschnikowia bicuspidata*, and illness‐mediated feeding behaviors (Strauss et al., 2019). For instance, infection prevalence varied from ∼10% to 50% and some genotypes reduced food intake up to ∼78% (Strauss et al., 2019).

These findings and unique aspects of this host–pathogen system (detailed below) allow us to address the following questions: Do more susceptible genotypes exhibit the strongest or weakest changes in feed intake? Do these changes benefit the host, pathogen, both, or neither? In an extensive literature review (Hite, Pfenning, & Cressler, 2020), these questions emerged as important gaps in knowledge. Of course, we recognize the causality dilemma inherent in these questions; hosts could have higher infection rates *because* they alter food intake. Addressing this dilemma, however, requires further investigation that is beyond the scope of our study. Here, we lay the foundation for future work by quantifying changes in behavior, pathogen transmission potential and host fitness. We hope that this first step will help uncover key patterns to guide future experiments in this and other host–pathogen systems with greater molecular resolution to identify more mechanistic and causal pathways.

The focal host–pathogen system is unique in that (1) transmission is relatively well understood (Ebert, 2005; Hall et al., 2007; Stewart Merrill & Caceres, 2018) and can be readily re‐created in the lab; (2) *Daphnia* are facultative parthenogens and typically reproduce asexually, and different isoclonal lines (hereafter: genotypes) often vary in key epidemiological traits like exposure (i.e. foraging rate) and per‐spore susceptibility (Auld et al., 2013; Hall et al., 2010); (3) pathogen fitness can be easily approximated by quantifying spore yield; and (4) host fitness and other covarying life‐history traits can be tracked over the entire course of infection.

Measuring these four intertwined parameters enables us to discriminate between resistance—the ability to control pathogen exposure, growth, or transmission—and tolerance—the reduction in infection‐induced pathology or fitness costs (Read et al., 2008; Schneider & Ayres, 2008). Quantifying this collection of traits in the same study presents notable logistical challenges (Graham et al., 2011) and is understandably missing from most studies on changes in feeding behaviors (Hite, Pfenning, & Cressler, 2020).

We find that infection‐mediated changes in host feeding behavior—which are traditionally interpreted as immunopathology—may in fact serve as crucial components of host defense strategies. Our results validate a protective effect of altered feeding behavior during pathogen exposure while also revealing significant variation in the magnitude of this response across host susceptibility and pathogen exposure levels. Across all host genotypes, feeding rates were negatively correlated with susceptibility to infection and transmission potential. More susceptible genotypes exhibited the strongest changes in feeding behavior.

## 2 STUDY SYSTEM

The *Daphnia–M. bicuspidata* system presents a rare opportunity to show how infection‐mediated feeding behaviors influence multiple traits of the host as well as pathogen fitness across pathogen exposure levels, and how these patterns vary across host genetic backgrounds. The focal hosts, *D. dentifera*, are key consumers in aquatic food webs throughout northern temperate lakes where they are hosts to numerous pathogens, including the fungal pathogen studied here, *M. bicuspidata*. Hosts become infected while filter‐feeding on phytoplankton. Upon successful infection, the fungal spores pierce through the host's gut wall into the body cavity and avoid degradation by host hemocytes (Metschnikoff, 1884; Stewart Merrill & Caceres, 2018).

This “obligate‐killer” pathogen exhibits a parasitoid life‐history strategy that requires the death of its host for onward transmission. However, before the pathogen can kill the host and release spores into the water column where they infect new zooplankton hosts, it must escape several innate immune responses. *Daphnia* have a relatively simple, albeit powerful, innate immune response. These responses include phagocytosis by hemocytes (Metschnikoff, 1884; Stewart Merrill & Caceres, 2018), phenoloxidase (Mucklow & Ebert, 2003), and changes in feeding behaviors (Hite et al., 2017; Strauss et al., 2019). In this study, we focus on changes in feeding behaviors, which mediate pathogen exposure (Ebert, 2005; Hall et al., 2007), tolerance, and multiple mechanisms of resistance (Hite, Pfenning, & Cressler, 2020), with important implications for downstream immune functions (Adamo et al., 2010; Povey et al., 2014).

## 3 METHODS

### 3.1 Combined foraging and infection assay

**FIGURE 1:**
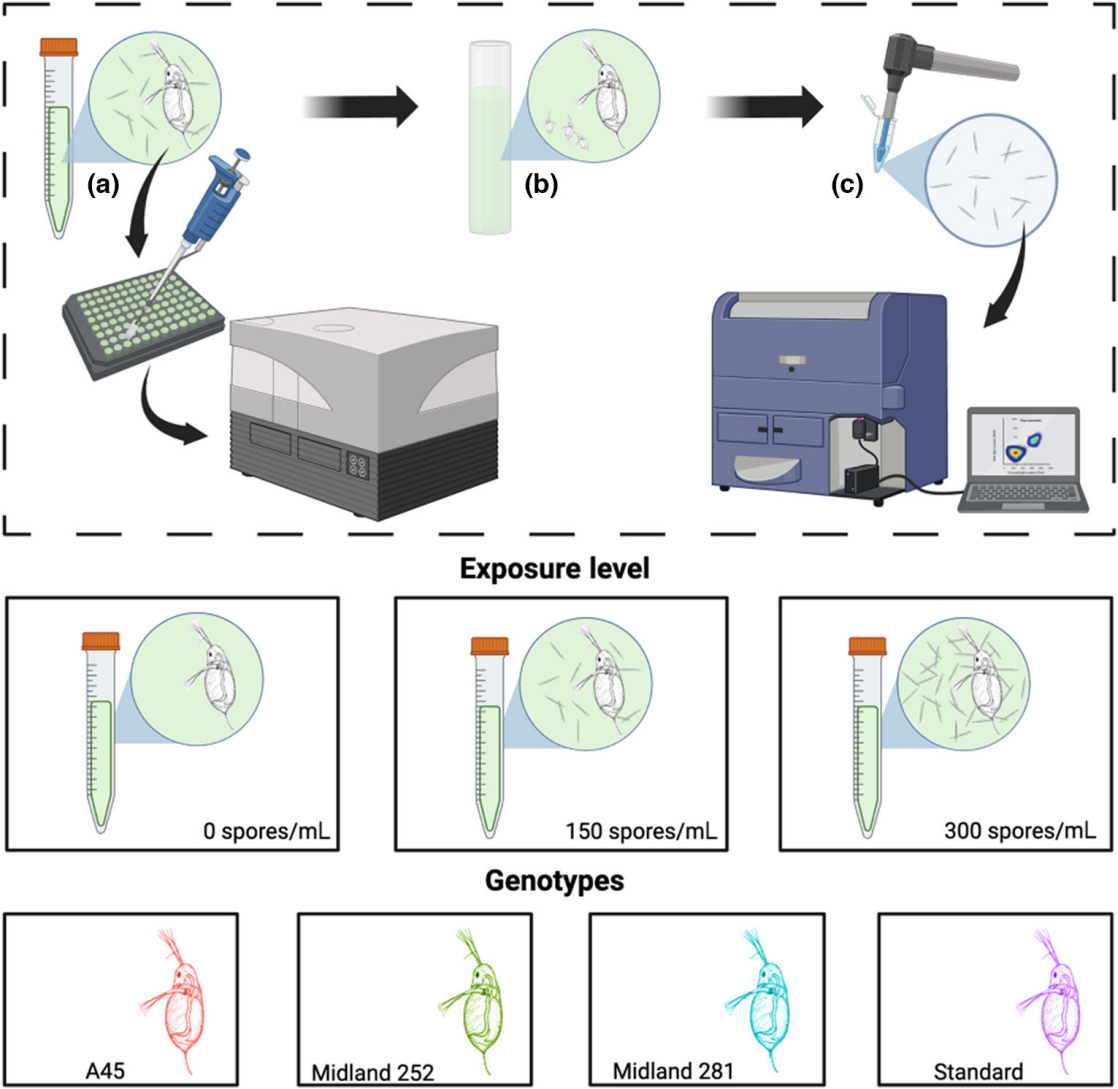
Experimental design to study links between susceptibility to infection and the net effects of changes in feeding behavior. (a) In a joint feeding‐rate infection assay, we tracked changes in feeding behaviors and susceptibility to infection across exposure levels (indicated in the figure by the density of small‐needle‐like spores). (b) We then examined several life‐history traits of the hosts (day of first reproduction and number of offspring produced each day) with a life table assay. (c) We then measured the final size of each individual, and used flow cytometry to quantify spore yield from the remaining infected individuals. To standardize the collection of spore yield, the experiment was terminated 14 days post‐exposure. This limits our ability to quantify effects of infection on host longevity. However, a central goal of this study was to examine effects on pathogen load at a biologically reasonable time point during infection.

We measured infection‐mediated feeding behavior in adult females (6‐day‐old) from four unique host genotypes across a gradient of three pathogen exposure levels (Figure 1; Hite et al., 2017; Strauss et al., 2019). All genotypes were chosen from existing cultures isolated from lakes in Michigan or Indiana (USA). Cultures were maintained in high nitrogen COMBO, artificial lake water media (Kilham et al., 1998), and fed lab‐cultured *Ankistrodesmus falcatus* (1 mgC/L). This alga resembles the shape (needle‐like) and size (40–50 μm long; 3–5 μm wide) of *M. bicuspidata* spores. Hosts were maintained at 22°C for at least three generations to standardize any maternal effects.

We cultured *M. bicuspidata* in vivo in a standard genotype of *D. dentifera*. We standardized all conditions known to affect the infection success of the pathogen, including temperature (Shocket et al., 2018), host age (Hite et al., 2017), algal quality and quantity (Hall et al., 2009), and spore age (Duffy & Hunsberger, 2019). Current evidence indicates that genetic differences among pathogen strains do not affect transmission success (i.e., infectivity and virulence, [Duffy & Sivars‐Becker, 2007; Searle et al., 2015; Shaw et al., 2021]). These results suggest that host traits predominately shape the outcomes of this host–pathogen interaction. All spores used in the infection assay were collected 21 days prior to the assay and counted with a hemocytometer.

We reared cohorts of neonates from each host genotype for 5 days (under a 16:8 light:dark photoperiod at 22°C). Then, individuals were isolated in vials (volume *V* = 10 mL) containing 1 mgC/L of *A. falcatus*. We inoculated 30 replicates of each genotype at each density of fungal spores: 0, 150, or 300 spores/mL (*N* = 360; 30 individuals × 3 spore treatments × 4 genotypes). We treated control vials identically to spore treatments, except that we did not add hosts. That is, controls contained algae and pathogen spores but no hosts to consume the algae or spores. To ensure that algae and spores remained suspended throughout the assay, we gently inverted all vials every 30 min. We conducted the entire assay (set‐up to take‐down) in the dark to prevent algal growth or any spurious spikes in algal fluorescence. Hosts were exposed to spores for 24 h, but we measured feeding rates after 7 h (based on J. L. Hite, unpublished data).

At the end of the 7‐h feeding rate assay, we collected subsamples from each vial to measure in vivo fluorescence following (Hite, Pfenning‐Butterworth, et al., 2020; Sarnelle & Wilson, 2008). In brief, this method compares the fluorescence of algae in vials with hosts vs. the fluorescence of algae in the host‐free (consumer‐free) controls. We measured algal fluorescence using narrow‐band fluorometry (Tecan, Maennedorf, Switzerland) with 200 μL in each well (each with two technical replicates) of a black 96‐well plate (14‐245‐197A Thermo Fisher Scientific No. 7605). For extended technical details, see Hite, Pfenning‐Butterworth, et al. (2020).

We calculated the feeding rate of individual hosts, *f*, following Sarnelle and Wilson (2008) and Hite, Pfenning‐Butterworth, et al. (2020), by solving for the change in fluorescence, *F*:

(1) $$\frac{dF}{dt}=\frac{-f}{V}F\left(t\right)$$

where *F*_*t*_ is the food remaining (the mean algal fluorescence of the sample at time, *t*, *F*_0_ is the initial amount of food‐the mean algal fluorescence of the corresponding plate‐specific animal‐free controls at time *t* = 0), *V* is the volume of media (10 mL), and *t* is the length of the assay. Solving for *f*, then:

(2) $$f=\ln\left(\frac{F_{0}}{F_{t}}\right)\frac{V}{t}$$

We omitted any negative feeding rates (since these represent technical errors and provide a more conservative estimate of infection‐mediated anorexia since strong negative feeding rates could bias the results), individuals that died during the assay, and animals identified as male (male and female *Daphnia* have different feeding rates, Hite et al., 2017).

### 3.2 Life‐history table

To examine the relationship between infection‐mediated feeding behaviors and host susceptibility, we measured the proportion of hosts that became infected and the survivorship of infected hosts relative to control hosts (0 sp/mL treatment). Then, to determine how changes in feeding behaviors affect the realized fitness of both hosts and pathogens, we quantified host growth, fecundity, and transmission potential (spore yield). We maintained individuals at 22°C in vials of 15 mL of COMBO for 14 days post‐exposure (or death, whichever came first). Individuals were moved to fresh media, fed (1 mgC/L for the first 7 days and 2 mgC/L for the rest), and checked for offspring every other day. To keep results consistent across individuals, we ended the experiment 14 days after exposure, when the first individuals began to die; our central goal here was to quantify changes in feeding rate and transmission potential (spore yield) and not longevity.

We visually diagnosed terminal infections with a dissecting microscope, measured final body size (at 5× magnification), and collected individuals to quantify spore loads using flow cytometry. We used a DxP10 flow cytometer (Cytek) equipped with a BD FACSort system (Becton Dickinson Biosciences). To isolate mature transmission‐ready spores from algae, animal debris, or immature spores (Stewart Merrill & Caceres, 2018), we used custom gates based on fluorescence forward scatter (FSC) and side scatter (SSC) with 488 and 561 nm lasers and fluorescent beads as standards (SPHERO; AccuCount Fluorescent Particles, 7.0–8.0 μm) at a ratio of 12:1 for each individual's spore solution (1 animal in 300 μL of COMBO). We then verified flow cytometry spore counts by randomly selecting five individuals from each genotype and spore exposure level and manually counting spores using a hemocytometer (for “Standard,” *R* ^2^ = .91; patterns for all other genotypes were comparable—see Appendix S1).

## 4 STATISTICAL ANALYSIS

All analyses were carried out in R statistical software. We used generalized linear models (GLMs) with host genotype, initial host size (mm), pathogen dose (0, 150, and 300), and their interaction as fixed effects. Frequency of infection was analyzed with binomial errors and log‐link function. Because feeding rates are log‐transformed, they were always analyzed with Gaussian distributed errors and either identity or log link functions. For survival, we conducted both time‐censored analyses and GLMs with longevity (time until death) as the response variable with Gaussian distributed errors and either identity or link functions. Both results were qualitatively similar (see Appendix S1). We present longevity in the main results because relative to the survival curves, they are easier to decipher (in our opinion). We ran saturated and reduced models and used Akaike information criteria (AICc) for model selection with limited sample size (R package: *MuMIn*). We then examined the selected models using the residual diagnostics, summary, and accessed significance using Wald chi‐square statistics (R package: *car*) following Fox, 2011. For post hoc analyses, we used least square means with Tukey's correction for multiple comparisons. The full code and statistical analyses are available together with the data on GitHub.

## 5 RESULTS

### 5.1 Susceptibility

**FIGURE 2:**
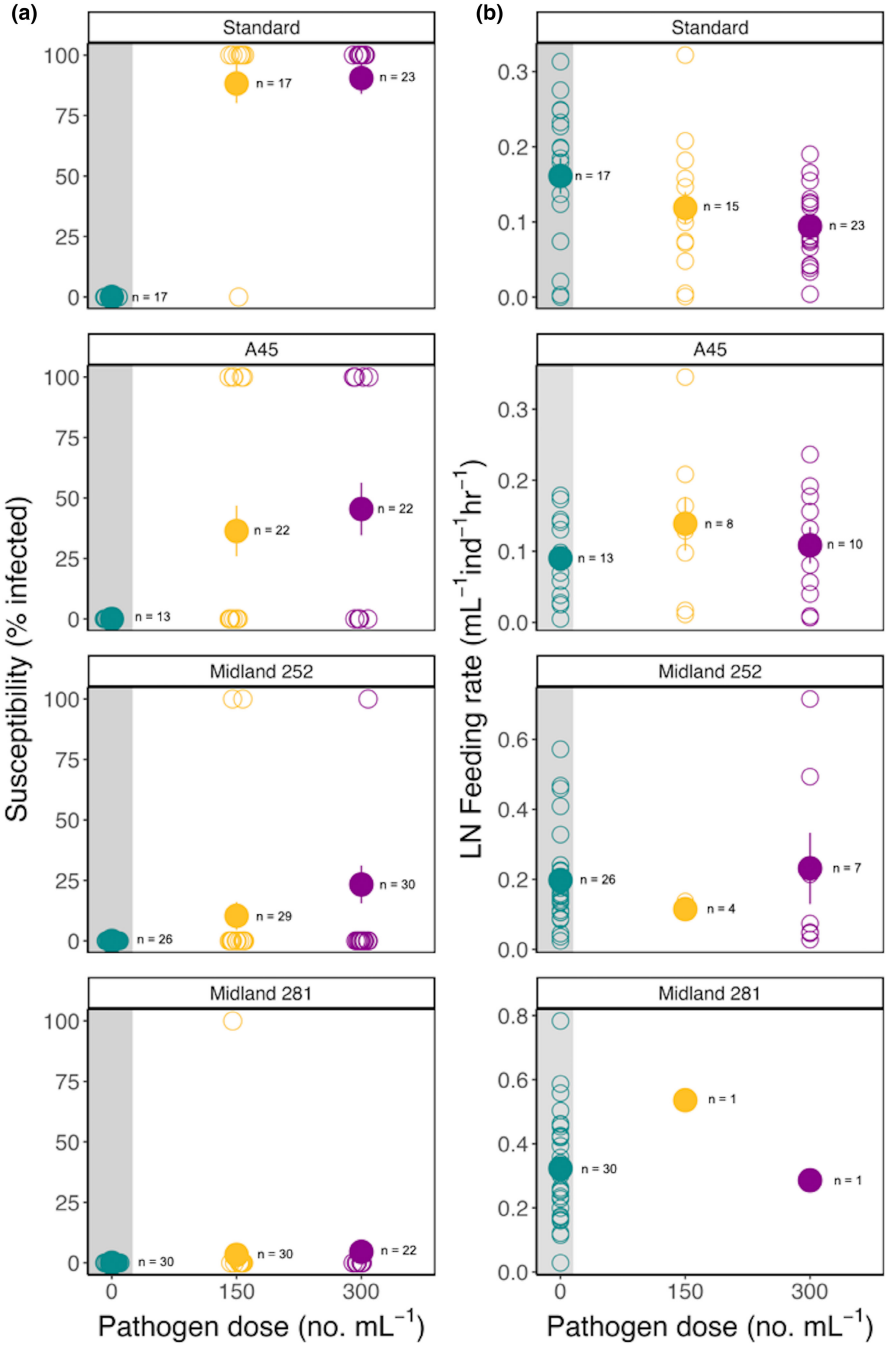
Natural variation in (a) host susceptibility to infection and (b) LN feeding rate of control and infected individuals as a function of pathogen exposure levels. Unfilled circles indicate replicates and filled circles indicate the mean ± standard error.

As expected, the focal genotypes varied in susceptibility to infection (Figure 2a). The “Standard” and “Midland 281” genotypes were the most and least susceptible genotypes, respectively. In the “Standard” genotype, all but one individual became infected at both pathogen doses. In the “Midland 281” genotype, only one individual was infected at both pathogen doses (because “Midland 281” only had one data point per pathogen exposure dose, it was not included in the statistical analyses). Relative to “Standard” and “Midland 281,” the other two genotypes exhibited intermediate levels of susceptibility. Infection frequency increased with pathogen dose across genotypes, but was not a statistically significant predictor of infection frequency (Table 1), even after accounting for differences in host body size which may impact exposure (feeding rate) and immune responses (see Appendix S1; Stewart et al., 2019).

**TABLE 1:** Factors influencing host susceptibility to infection (% infected). See Sections 3 & 4 for further details on infection assays and statistical analysis.

Term	Wald *χ* ^2^	df	Pr(>*χ* ^2^)
Genotype	18.75	2	**8.4 × 10** ^**−5**^
Pathogen exposure level	0.66	1	.416

*Note*: Bold indicates significance values (*p* < .05).

### 5.2 Feeding behavior

We observed pronounced phenotypic variation in the defense mechanisms of focal host genotypes. For example, analysis of total food consumption, a metric commonly used to quantify infection‐mediated anorexia and hyperphagia, showed that one genotype altered their feeding behavior in a dose‐dependent manner and the others did not (Figure 2b). For the “Standard” genotype, infected individuals decreased food intake by up to 42%, depending on pathogen dose (Figure 2b). On the other hand, infected individuals from the “A45” genotype increased food intake (hyperphagia) by up to 54% depending on pathogen dose (Figure 2b). For the “Midland 252” genotype, infected individuals either decreased food intake by approximately 42% at the intermediate spore dose or increased food intake by 17% at the high spore dose (Figure 2b). Together, these results indicate that for *Daphnia*, infection‐mediated anorexia and hyperphagia are both genotype and dose dependent (Table 2).

**TABLE 2:** Factors influencing host feeding behavior. See Sections 3 & 4  for further details on feeding rate assay and statistical analysis.

Term	Wald *χ* ^2^	df	Pr(>*χ* ^2^)
Genotype	56.68	2	**3.0 × 10** ^**−12**^
Initial body size (mm^2^)	17.10	1	**3.5 × 10** ^**−5**^
Pathogen exposure level	0.008	1	.929

*Note*: Bold indicates significance values (*p* < .05).

### 5.3 Net outcomes

#### 5.3.1 Linking host susceptibility to host and pathogen traits

Across all genotypes, susceptibility to infection and spore yield were positively correlated (*r* = .735, *t* = 15.99, *p* < .001, 95%, CI = (0.667, 0.790)). In other words, more susceptible genotypes tend to produce more spores, and therefore, have higher spore yields (and transmission potential), regardless of pathogen exposure levels. How did this genotypic variation in susceptibility and spore yield, in turn, affect host fitness, growth, and survival? For the most susceptible genotype (“Standard”), infection had no effect on host fitness (total reproduction; see Appendix S1). For all genotypes, host fitness was influenced by pathogen exposure level and the interaction between genotype and exposure level (Table 3). Infection was associated with either a slight increase (“A45”) or a slight decrease in reproduction (“Midland 252”; see Appendix S1). Infection also altered host growth (Table 3), especially for the most susceptible genotype, “Standard” (see Appendix S1). Finally, pathogen exposure levels did not significantly affect host survival (Table 3).

**TABLE 3:** Factors influencing host fitness (total offspring produced), host growth (final−initial size), and survival. See Sections 3 & 4 for further details on the life‐history table and statistical analysis.

Term	Wald *χ* ^2^	df	*p* Value
*Host fitness*			
Total Growth (mm^2^)	1.15	1	.282
Genotype	31.70	2	**1.3 × 10** ^**−7**^
Pathogen exposure level	9.09	1	**.003**
Genotype × Path. exp.	12.87	2	**.002**
*Host growth*			
Pathogen exposure level	11.18	1	**8.2 × 10** ^**−4**^
Genotype	19.92	2	**4.7 × 10** ^**−5**^
*Host survival*			
Pathogen exposure level	3.38	1	.066
Genotype	2.10	2	.349

*Note*: Bold indicates significance values (*p* < .05).

#### 5.3.2 Linking feeding behaviors to host and pathogen traits

**FIGURE 3:**
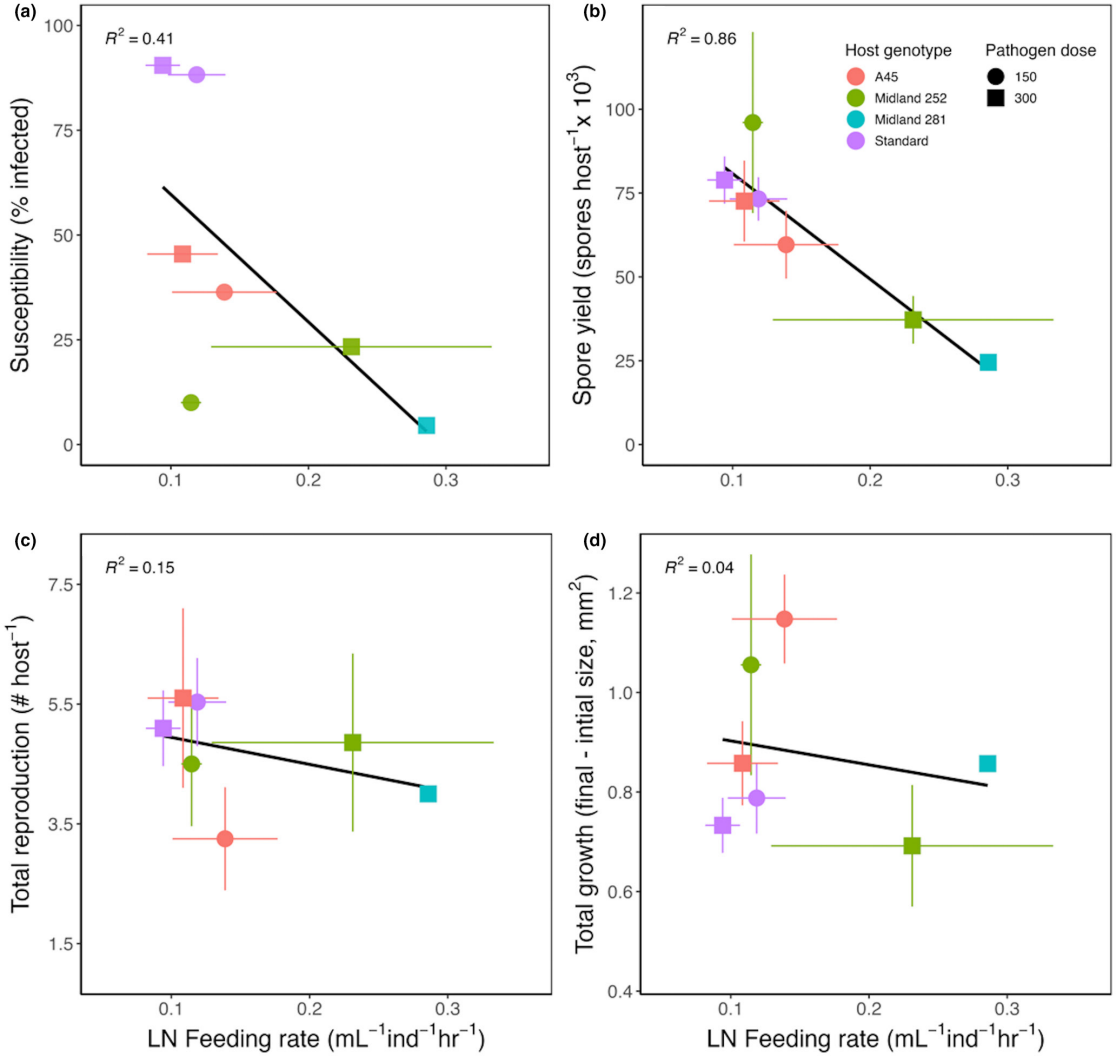
Connecting variation in feeding behavior to key host and pathogen traits. (a) More susceptible genotypes tend to reduce food intake when infected and (b) produce more spores, a key metric of pathogen fitness. (c) Reduced feeding behavior tends to increase fecundity and (d) growth. Symbols indicate mean ± standard error of infected individuals. Black line indicates linear regression across genotypes and pathogen doses; multiple *R* ^2^ is indicated for each regression.

Across all four host genotypes, feeding rates were negatively correlated with susceptibility to infection, transmission potential (GLM: *χ* ^2^ = 18.57, *p* < .001, Figure 3a), and pathogen loads (spore yield) (GLM: *χ* ^2^ = 17.29, *p* < .001, Figure 3b). Notably, the most susceptible genotype (“Standard”), which produced higher spore yields across pathogen doses, was also one of the smallest of the genotypes (Figure 3d). This genotype, therefore, produced high spore yields and maintained reproductive levels similar to its uninfected counterparts (see Appendix S1) despite drastically reducing food intake (which should have also decreased ingestion of pathogen spores) and suffering reduced growth rates (Figure 3d). Such pronounced differences in this genotype may help explain why, after accounting for genotypic differences, the effects of feeding rates and host size (mm^2^) on susceptibility were not statically significant (Table 4b).

**TABLE 4:** (a) Factors influencing host susceptibility (% infected) and (b) transmission potential (spore yield) across host genotypes.See Sections 3 & 4  for further details on infection assays and statistical analysis.

Term	Wald *χ* ^2^	df	Pr(>*χ* ^2^)
(a)			
*Susceptibility to infection (% infected)*			
Feeding rate	1.24	1	.265
Initial body size (mm^2^)	0.65	1	.421
*Transmission potential (spore yield)*			
Pathogen exposure level	0.84	1	.361
Final body size (mm^2^)	6.23	1	**.013**
(b)			
*Susceptibility to infection (% infected)*			
Feeding rate	0.19	1	.660
Initial body size (mm^2^)	0.29	1	.591
Pathogen exposure level	0.58	1	.445
Genotype	16.83	3	**.0002**
*Transmission potential (spore yield)*			
Genotype	3.77	2	.152
Pathogen exposure level	1.01	1	.315
Final body size (mm^2^)	7.85	1	**.005**

*Note*: Bold indicates significance values (*p* < .05).

## 6 DISCUSSION

Understanding how variation in infection‐mediated changes in feeding behaviors influence host disease and pathogen transmission is essential if we are to develop effective immunotherapies, identify pathologies driven by the host or the pathogen, and develop a deeper understanding of the physiological and behavioral underpinnings of host defense (Ayres & Schneider, 2009; Cumnock et al., 2018; Kyriazakis et al., 2009). As a step toward addressing this puzzle, we set out to test two hypotheses: (1) immune‐mediated anorexia is an avoidance tactic that helps hosts to reduce encounters with orally transmitted pathogens (anti‐infection resistance), (2) hyperphagia is immune enhancing and therefore limits pathogen growth. By examining these behaviors across genetically diverse hosts collected from nature, we find mixed support for both hypotheses, while highlighting the pronounced variation in the magnitude of this response across host susceptibility and pathogen exposure levels.

Do more susceptible genotypes exhibit the strongest or weakest anorexia? Across all four host genotypes, feeding rates were negatively correlated with susceptibility to infection and transmission potential. Overall, more susceptible genotypes exhibited the strongest changes in feeding behavior, but these results varied with pathogen exposure levels. The most susceptible genotypes exhibited either strong anorexia, reducing food intake by ∼26%–42% (“Standard”), or pronounced hyperphagia, increasing food intake by ∼20%–54% (“A45”). In contrast, the least susceptible genotype showed no change in feeding rate, regardless of pathogen exposure level. These results join other studies indicating that the magnitude of immune responses appears finely tuned to the severity of the threat. In sheep, more susceptible host genotypes also appear to exhibit stronger anorexia than their less susceptible counterparts (Zaralis et al., 2008). While fewer studies have measured the dose–response of hyperphagia (see Hite, Pfenning, & Cressler, 2020 and references therein), anorexia appears to increase with higher levels of parasite exposure or parasitemia, as seen, for example, in fish (de Jesus et al., 2018; Li & Woo, 1991; Pirhonen et al., 2000), sheep (Coop et al., 1982; Kyriazakis et al., 2009), frogs (DeMarchi et al., 2015), rabbits (Nakamura et al., 1994), and zooplankton (Hite et al., 2017; Narr & Frost, 2015).

Do changes in infection‐mediated feeding behaviors benefit the host, pathogen, both, or neither? Answering this question requires understanding how changes in feeding influence both host and pathogen fitness. This goal is complicated by logistical constraints, suffers from the causality dilemma, and is, understandably, missing from most studies on this topic (but see Ayres & Schneider, 2009; Rao et al., 2017; Rogers & Bates, 2007). However, the unique aspects of the focal host–pathogen system studied here enabled us to begin addressing this question across a small sample of genetically diverse hosts.

Changes in feeding rate were most pronounced for the most susceptible genotype, “Standard.” For this genotype, decreased food intake appears to help limit pathogen‐induced pathology (i.e., tolerance). That is, while the reduction in food intake did not suppress pathogen growth, it may have helped to offset the fecundity costs of infection, despite the reduced growth rates and smaller size of individual hosts. These results join others in demonstrating that anorexia can function as a tolerance mechanism (Ayres & Schneider, 2009; Cumnock et al., 2018), but likely varies with genotypic variation in susceptibility. From an evolutionary epidemiology perspective, tolerance mechanisms typically lead to the evolution of higher virulence (Budischak & Cressler, 2018; Carval & Ferriere, 2010; Restif & Koella, 2003). From an applied perspective, these results suggest that when anorexia increases tolerance, genotypes exhibiting strong anorexia may fuel both larger and more severe epidemics.

For the other genotypes, changes in feeding rates were more variable, marginally increasing or decreasing, depending on pathogen exposure levels. Two genotypes (“A45” and “Midland 281”) exhibited hyperphagia at intermediate spore doses. However, with “Midland 281,” only two individuals became infected, and therefore, links with feeding rates are challenging. These two genotypes highlight other interesting contrasts with the more tolerant (“Standard”) genotype. Interestingly, “Midland 281” had much lower infection prevalence, despite higher feeding rates, which should have increased exposure to these orally transmitted pathogens. Thus, a key open question is which immune mechanisms help counter such high pathogen exposures. For “A45,” with intermediate susceptibility levels, fitness (total reproduction) was lower when uninfected, and tended to increase at higher pathogen loads. These results are consistent with life‐history theory (Stearns, 1992) where stress responses lead to higher reproductive effort. While teasing apart these various mechanisms is beyond the scope of the current study, these results join mounting evidence highlighting that, beyond immune cells, defense against infectious agents involves an integrated and adaptive plastic arsenal of behavioral, physiological, and metabolic changes (Abu Kwaik & Bumann, 2013; Cotter et al., 2019; Cumnock et al., 2018; Cunrath & Palmer, 2021).

While the epidemiological and evolutionary importance of illness‐mediated feeding behaviors is increasingly appreciated (Cotter et al., 2019; Pike et al., 2019; Smith et al., 2015; Smith & Holt, 1996), previous studies have largely focused on the molecular, physiological, or immunological underpinnings in single host genotypes (Adamo et al., 2007; Adelman & Martin, 2009; Bashir‐Tanoli & Tinsley, 2014) or genetically modified model systems (Rao et al., 2017; Schneider & Ayres, 2008). Further, these host‐centric studies rarely track changes in host or pathogen life‐history traits (reviewed by Hite, Pfenning, & Cressler, 2020). As a consequence, the potential effects of illness‐mediated feeding behaviors on host fitness and pathogen transmission remain poorly resolved. Our results underscore that efforts to evaluate when and why immune‐mediated behaviors cause more harm to the host (immunopathology) or the pathogen, must consider the effects on multiple host traits and pathogen fitness, especially in light of the immense genetic diversity for host immune defenses (Duffy et al., 2021; Graham et al., 2011; Mucklow et al., 2004).

This study also highlights the inherent challenges associated with understanding genotypic variation in host susceptibility. Yet, if we consider that ecology is founded on and propelled by the exciting goal of understanding the immense genetic diversity found in natural systems, these results reconfirm the need to move beyond non‐model organisms to expand our understanding of immune polymorphism found in nature (Duffy et al., 2021; Graham, 2021). Such efforts require a greater appreciation for the fact that defense strategies fall along a spectrum that cannot be neatly categorized as resistance or tolerance. Of course, these efforts will require the development of molecular tools for non‐model organisms. We do not underestimate the logistical challenges associated with such endeavors, but rather join recent studies emphasizing the need for these tools and collaborations. In the meantime, approaches such as ours that combine tools from population ecology and immunology provide valuable insight into how natural selection shapes strategies for host defense and pathogen transmission.

## AUTHOR CONTRIBUTIONS

**Alaina C. Pfenning‐Butterworth:** Conceptualization (equal); formal analysis (equal); investigation (equal); writing – original draft (equal); writing – review and editing (equal). **Rachel E. Vetter:** Investigation (equal); writing – review and editing (supporting). **Jessica L. Hite:** Conceptualization (equal); formal analysis (equal); writing – original draft (equal); writing – review and editing (equal).

## Supporting information


Appendix S1

## Data Availability

All data and R code are publicly available from the GitHub repository (https://github.com/alainapb/Tolerance_Resistance).
