# Supplementary material for: Natural variation in host feeding behaviors impacts host disease and pathogen transmission potential
Source: Ecol Evol. 2023 Mar 7;13(3):e9865. doi: 10.1002/ece3.9865 (PMC9992943; doi:10.1002/ece3.9865)
Supplement: Supplementary file 1 — Appendix S1 [file ECE3-13-e9865-s001.pdf]

Appendix

APB

7/23/2022

Comparison of spore counts using flow cytometry to traditional counts using a hemocytometer in 'Standard' genotype

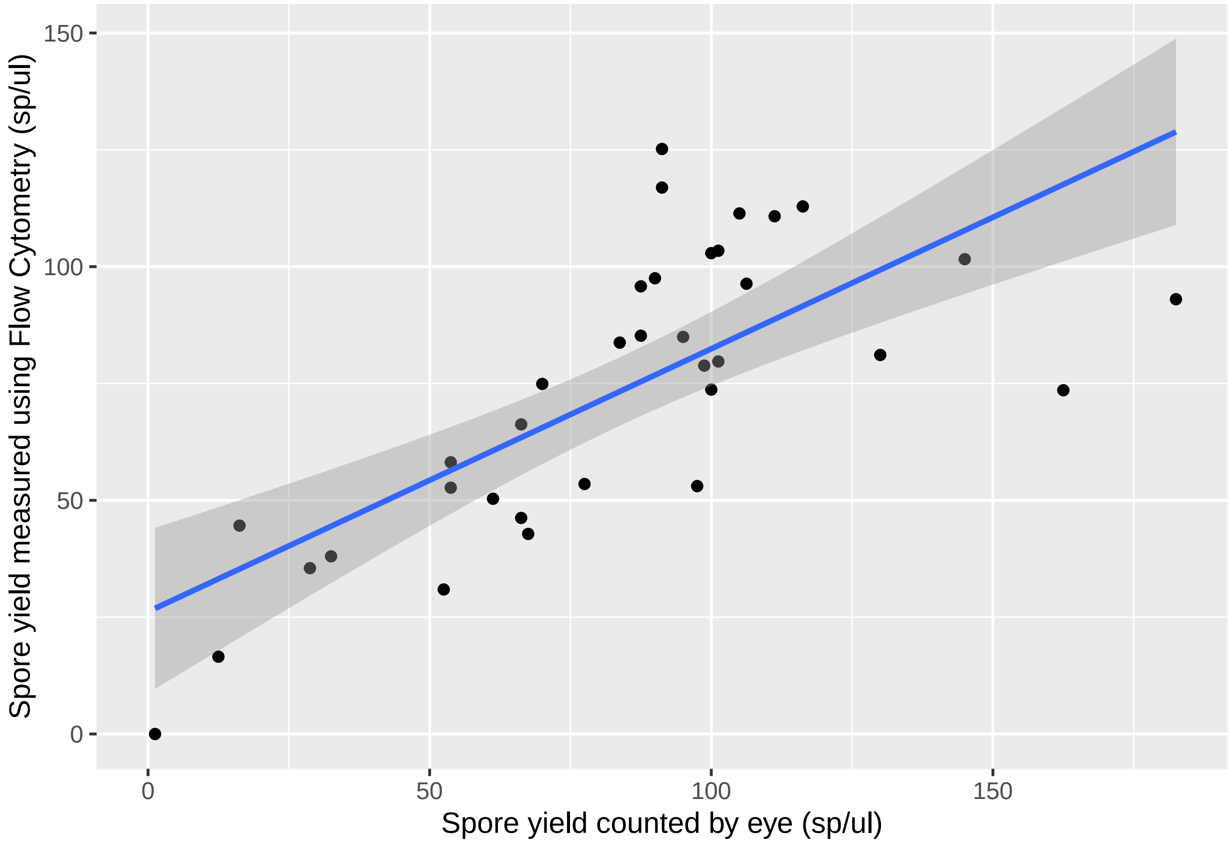


```
fit<-lm(sp.ul ~ flow_sp_ul - 1, data = sporecheck)
# R squared
summary(fit)$r.squared
```

```
## [1] 0.9130979
```

Host genotypes differ in susceptibility and transmission potential (spore yield)

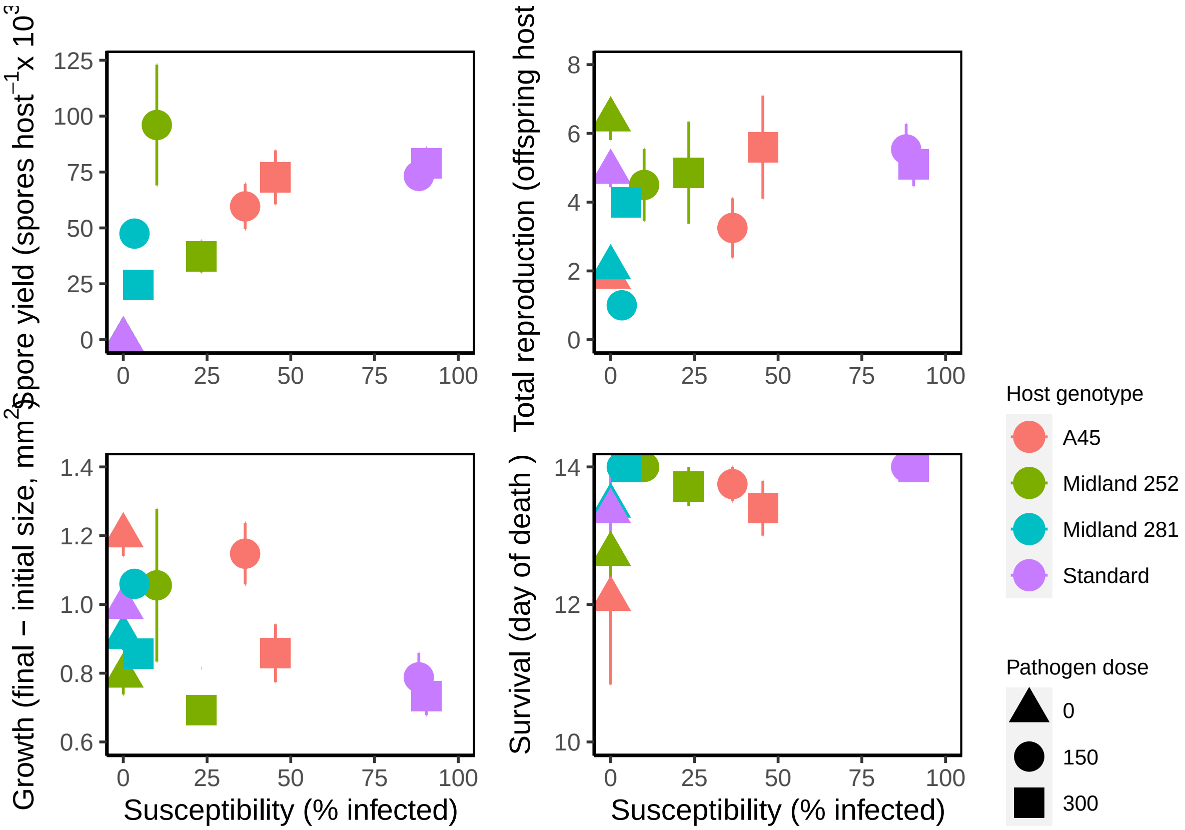

Impacts of Feeding Behavior

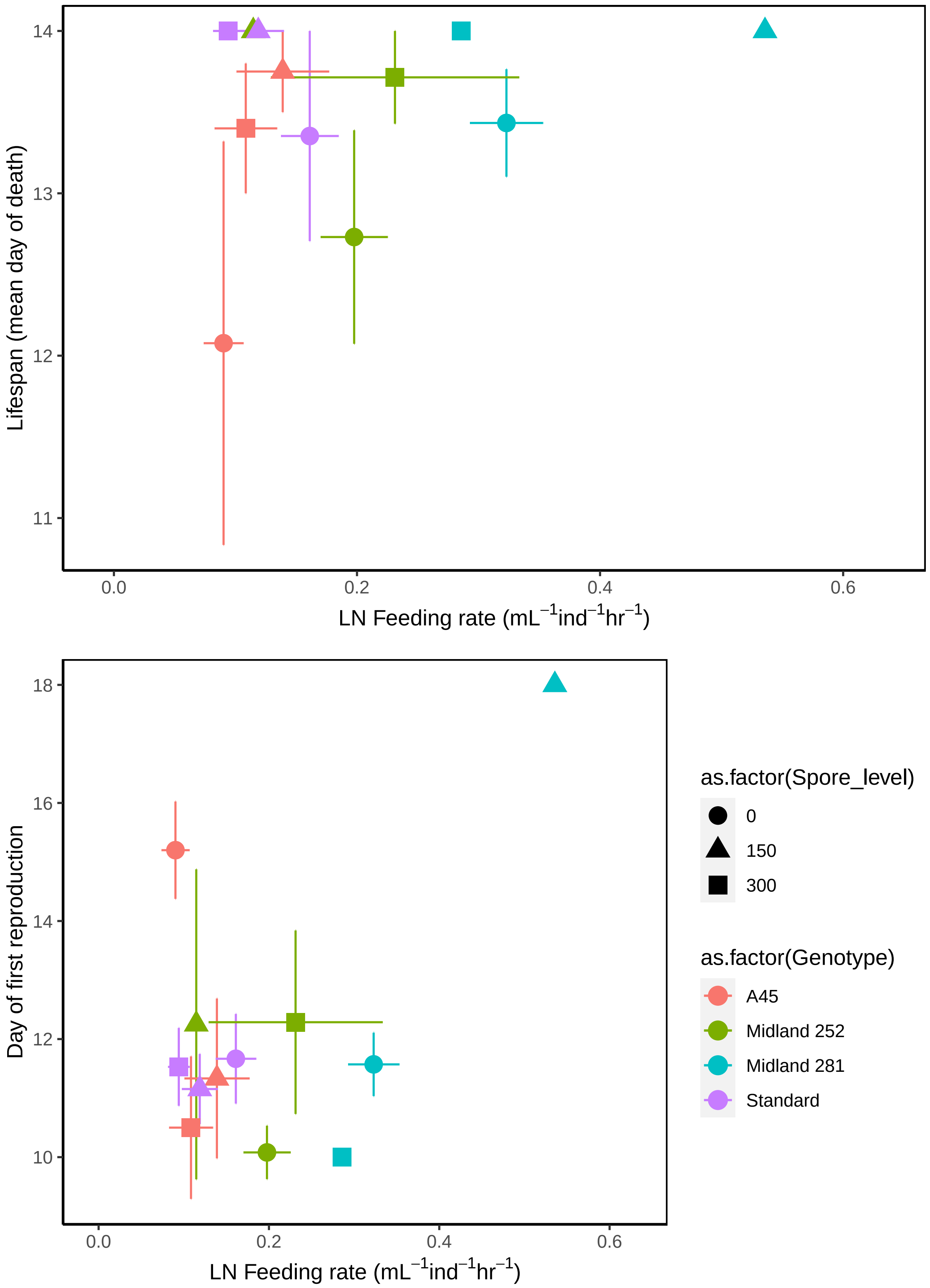

Impacts of Infection

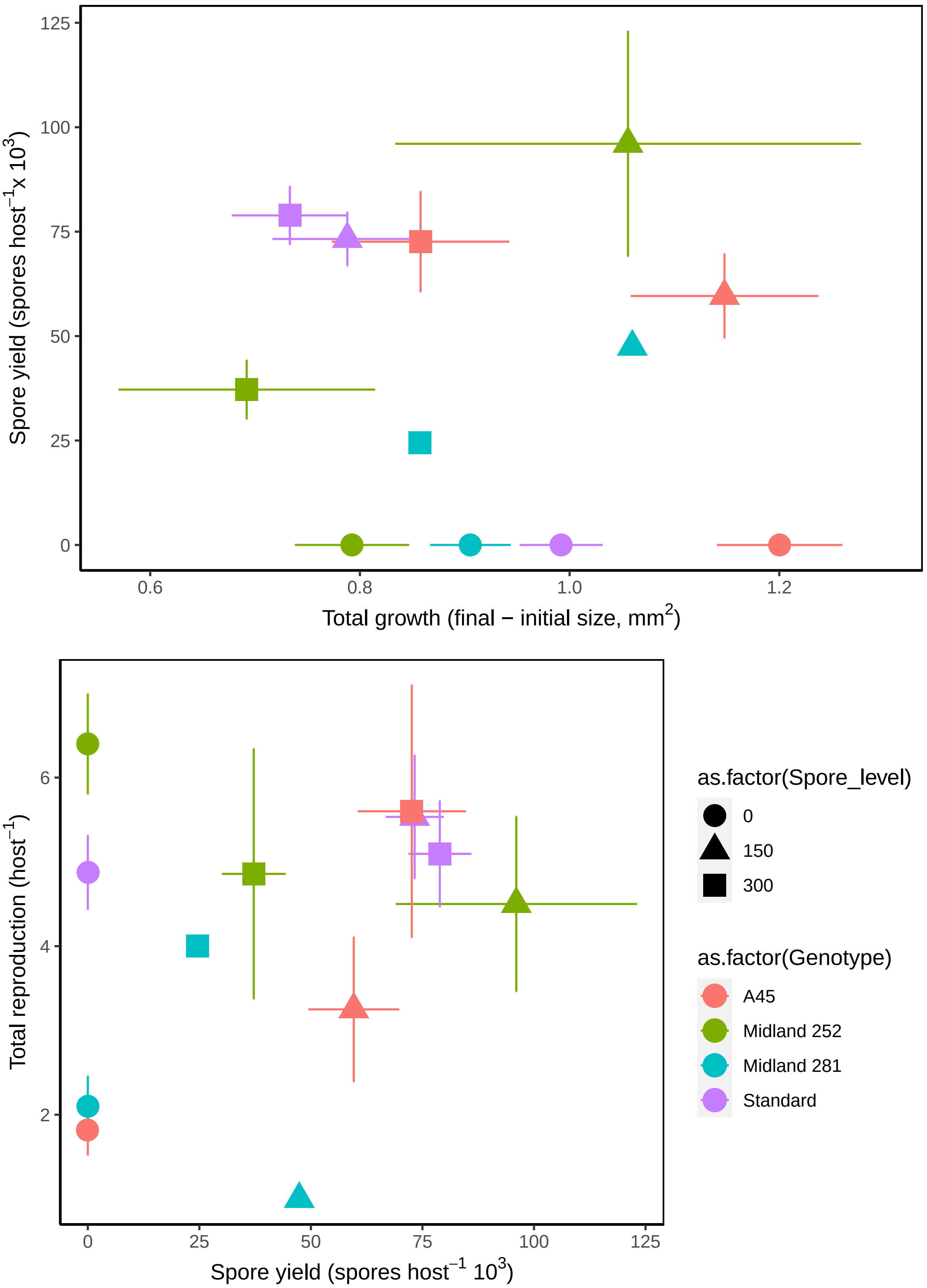

Survival Analyses

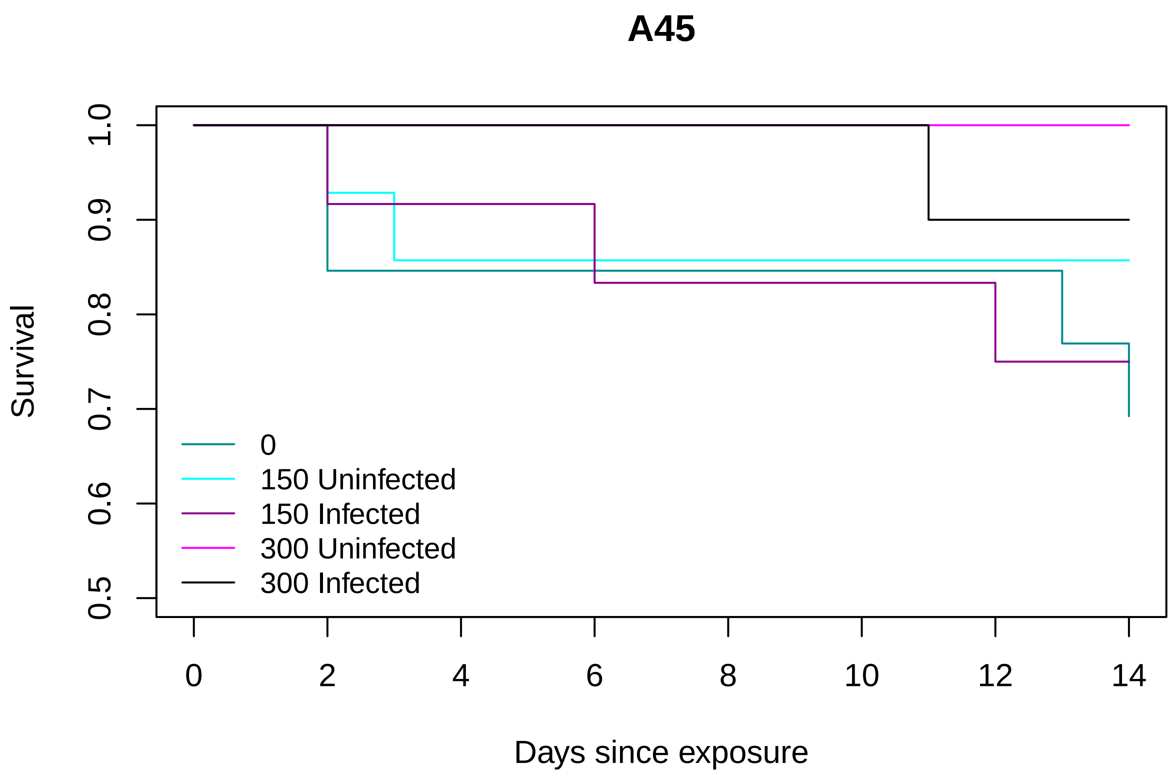

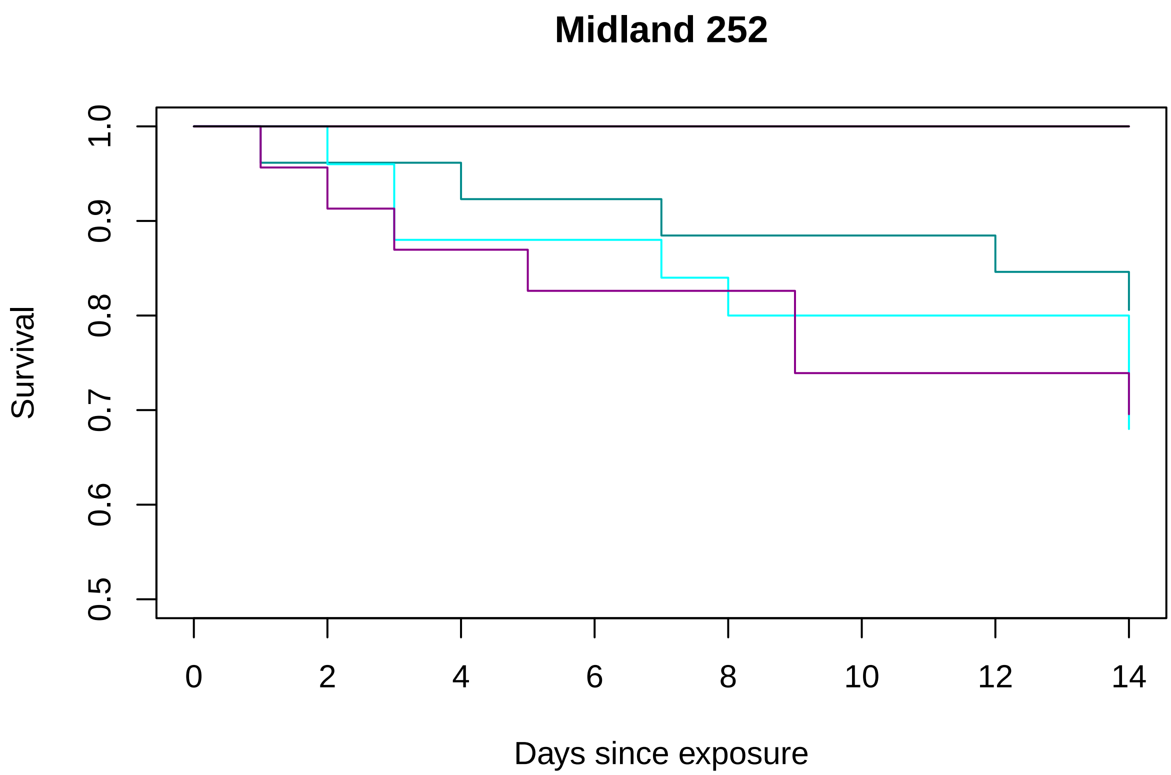

Survival Analyses Continued

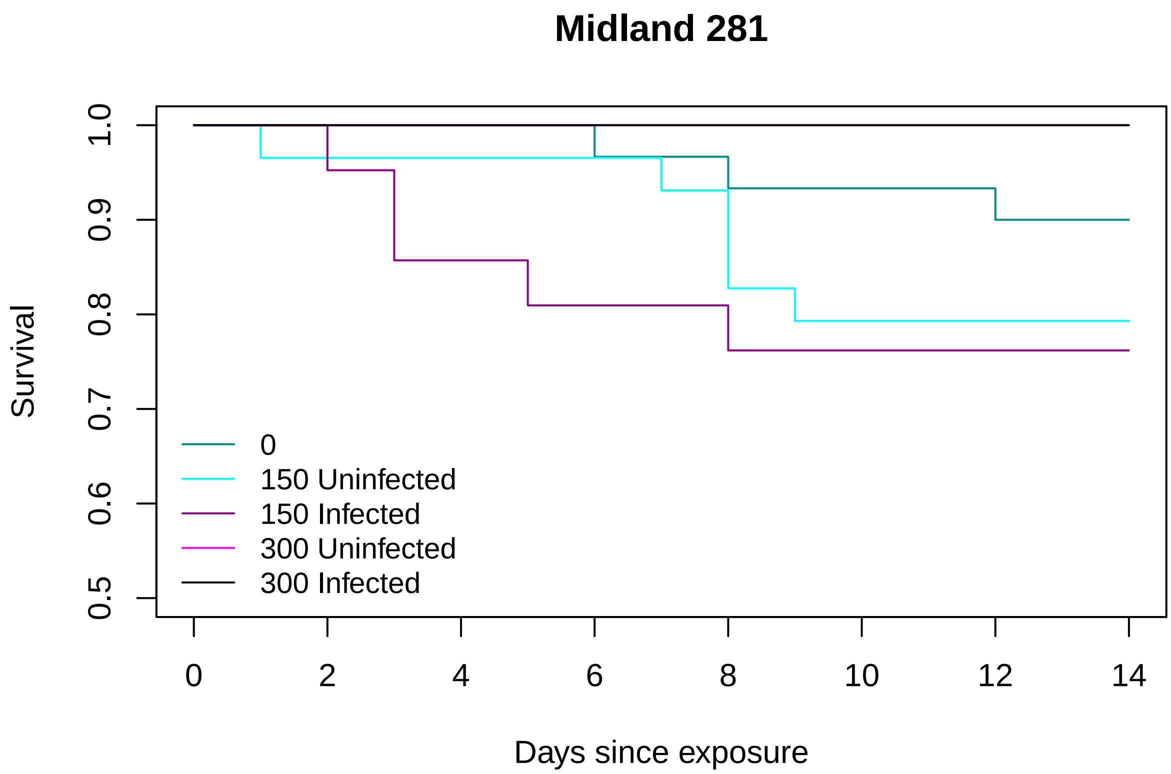

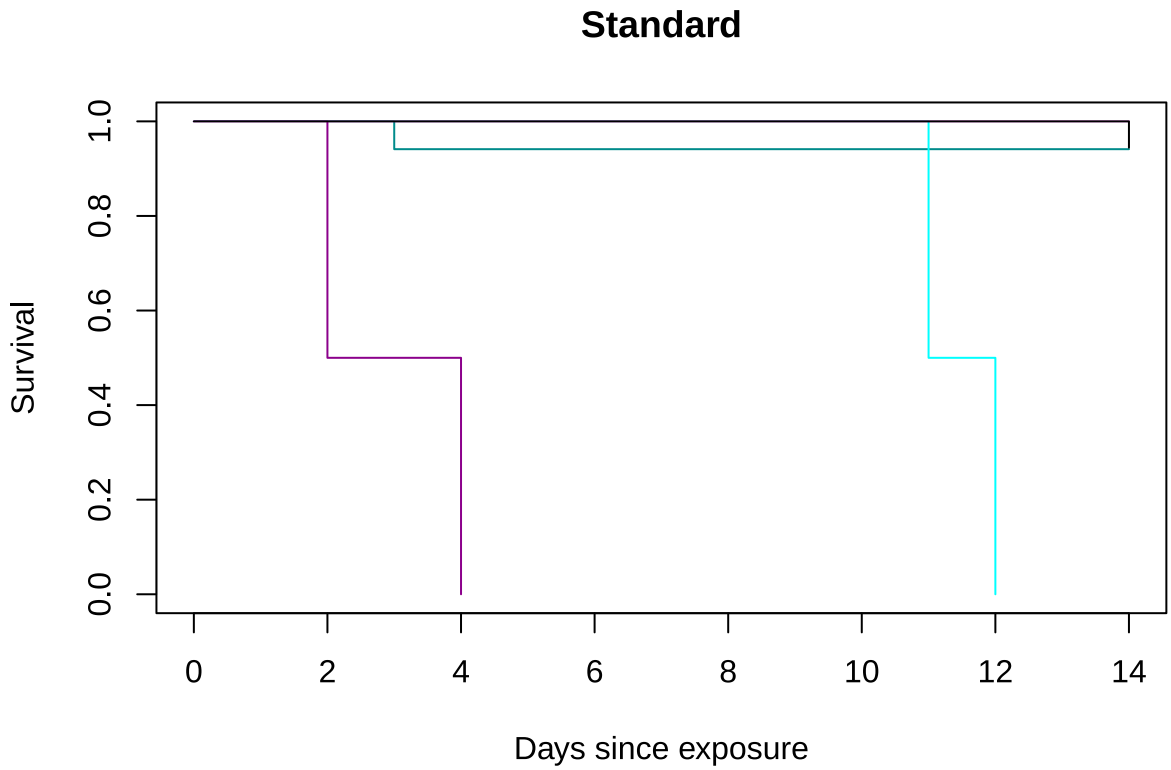

In Standard, there was only one individual that was exposed to 150 sp/ml that did not get infected and that individual died eleven days after the exposure (blue line).
